# Meta-analytic evidence for the complex mechanisms underlying congruency sequence effect

**DOI:** 10.1007/s00426-025-02093-5

**Published:** 2025-03-06

**Authors:** Yunji Lee, Paul Verhaeghen, Eliot Hazeltine, Eric H. Schumacher

**Affiliations:** 1 School of Psychology, Georgia Institute of Technology, Atlanta, USA; 2 Department of Psychological and Brain Sciences, University of Iowa, Iowa City, USA

## Abstract

**Introduction:**

The congruency sequence effect (CSE) refers to a reduction in the congruency effect after incongruent trials compared to congruent trials in a conflict-inducing task. There is an ongoing debate about the mechanisms underlying the CSE.

**Methods:**

To help inform this debate, we conducted a meta-analysis of the relevant CSE studies published in the past 31 years (from 1992 to 2023). By measuring the mean effect sizes from various tasks and procedures, we examined to what extent the CSE results from top-down or bottom-up mechanisms and to what extent these mechanisms are local to the tasks performed or global to the experiment.

**Results:**

Results demonstrate that while the CSE was larger for studies that included bottom-up confounds (Hedges’ *g* = 1.28), it was still robust and significant for studies that controlled for these confounds (Hedges’ *g* = 0.95). Additionally, CSE was significant both within (Hedges’ *g* = 1.54) and between tasks (Hedge’s *g* = 0.27), but the effect was larger within a task. This suggests that the mechanisms driving the CSE affect both local and global control mechanisms. Furthermore, the current meta-analysis suggests that the congruency effect and CSE may not result from the same control mechanisms. Lastly, given that bottom-up confounds are easily controlled for in the prime probe and temporal flanker tasks, which produced a large CSE (Hedges’ *g* = 1.13), these may be useful procedures to use to address future questions for CSE.

**Conclusion:**

Overall, the present meta-analysis provides converging evidence for conclusions from previous studies of the CSE and highlights the complex factors that produce this effect.

**Supplementary Information:**

The online version contains supplementary material available at 10.1007/s00426-025-02093-5.

## Introduction

Cognitive control is the ability to integrate information from the environment and internal drives to produce actions that achieve our goals. Cognitive control involves many processes, including selective attention, response inhibition, task switching, error monitoring, and conflict resolution (Miller & Cohen, 2001; Miyake et al., 2000). Experimental tasks are unlikely to exclusively engage a single cognitive process; rather, they involve multiple cognitive control processes. Therefore, it may be difficult to fully understand how each cognitive control function influences the experimental results in a single study. Also, the behavioral measurements from cognitive task, such as conflict effects, are notoriously noisy and heterogeneous (Enkavi et al., 2019; Hedge et al., 2018). A meta-analysis, which examines the effect sizes of many studies grouped by theoretically relevant experimental factors, may provide a more comprehensive understanding of an effect by isolating factors common across a broad range of studies.

### Conflict tasks and congruency sequence effect

One popular way to study cognitive control is with conflict tasks that measure performance differences in reaction times (RTs) and accuracy between congruent and incongruent trials. Three commonly used conflict tasks are the Stroop task (MacLeod, 1991; Stroop, 1935), flanker task (Eriksen & Eriksen, 1974), and Simon task (Simon, 1969). In the color-naming Stroop task, participants are required to name the ink color of a color-word (e.g., RED). The ink color can be either congruent (red) or incongruent (e.g., green). Incongruency between the ink color and the meaning of the word leads to slower RTs compared to the congruent trials (MacLeod, 1991). A spatial Stroop task requires participants to respond to the spatial location of a stimulus while ignoring incongruent spatial information, such as a left-pointing arrow presented on the right side (MacLeod, 1991). In the traditional flanker task, the stimulus consists of strings of letters. Participants are required to respond to the center target letter (e.g., left button press to S, right button press to H). The surrounding flanker letters can be congruent (e.g., SSSSS) or incongruent (e.g., HHSHH) with the target letter. In the Simon task, participants make responses based on a stimulus (e.g., left or right button presses to a red or blue square). The stimuli are presented either to the left or right of fixation. Participants respond to the stimulus with the assigned responses and conflict arises from the incongruency between the location of the stimulus and response, resulting in slower RTs compared to congruent trials.

A fourth type of conflict task, the prime-probe task, has become popular recently because it separates the presentation of the distracting and task relevant stimulus. In the prime-probe task, participants are presented with two stimuli in succession and are required to ignore the first stimulus (i.e., the prime) and respond only to the second target (i.e., the probe). For example, Kunde and Wühr, (2006) used a prime-probe arrow task, in which a relatively small distractor arrow preceded a relatively large target arrow. In this task, the prime and probe may be congruent (e.g., indicate the same direction) or incongruent (e.g., indicate different directions). A similar task, referred to as the temporal flanker task because both the first and second stimulus come from the same set (e.g., are the same size, modality, etc.), also produces robust congruency effects (Hazeltine et al., 2011; Schumacher et al., 2011; Weissman et al., 2015, 2020).

**Fig. 1:**
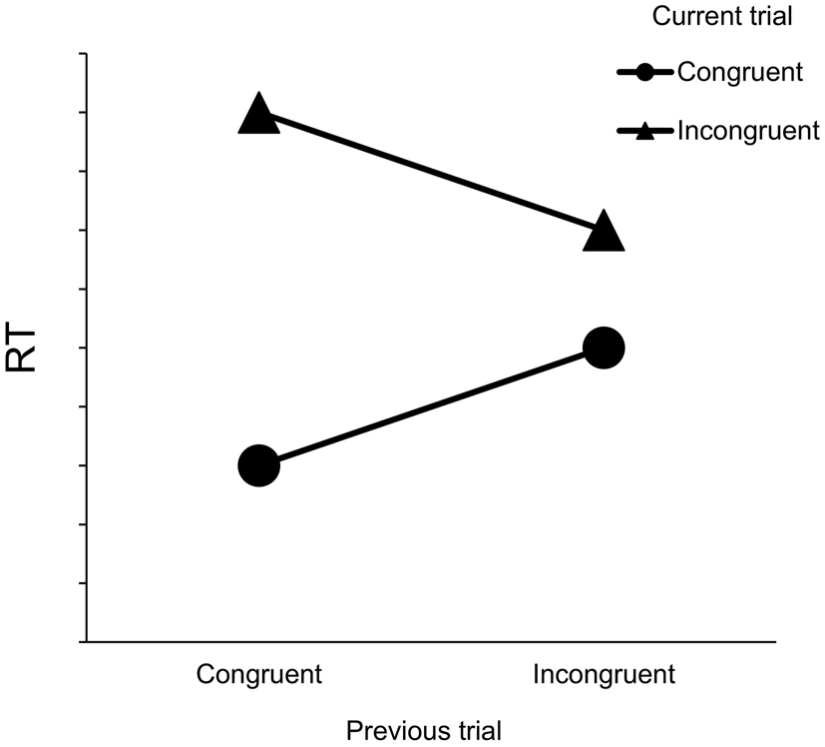
Schematic depiction of the congruency sequence effect (CSE) in response time (RT). The typical pattern shows an interaction between the size of the congruency effect and the congruency status of the previous trial

Researchers have used these conflict tasks to study not just conflict within a trial but also to measure the dynamics of cognitive control – that is, how control changes or adjusts across trials. Performance is affected by the congruency of the current trial, but this effect is moderated by the congruency of the previous trial. This phenomenon, which was first reported by Gratton et al. (1992), is referred to as the congruency sequence effect (CSE, also known as the Gratton effect, conflict adaptation effect, and the sequential modulation effect). In conflict tasks, performance on incongruent trials preceded by incongruent trials (iI) is faster than on incongruent trials preceded by congruent trials (cI) and performance on congruent trials following incongruent trials (iC) is slower than in those following congruent trials (cC) (for a review; Egner, 2007). Figure 1 shows the general RT pattern for the CSE often found in the literature (Clayson & Larson, 2011; Egner & Hirsch, 2005; Gratton et al., 1992; Kim & Cho, 2014; Notebaert et al., 2006; Ullsperger et al., 2005; Weissman et al., 2015).

In conflict tasks, performance on incongruent trials following incongruent trials (iI) are faster than on incongruent trials following congruent trials (cI) and performance on congruent trials preceded by incongruent trials (iC) are slower than on those preceded by congruent trials (cC).

### Accounts for congruency sequence effect

Two classes of accounts have been proposed to explain the CSE. One class is based on top-down control and the other proposes that the CSE is a byproduct of associative bottom-up processes. One of the most influential top-down control accounts, conflict monitoring theory, holds that conflict triggers a top-down modulation of attentional control (Botvinick et al., 2001, 2004). According to the conflict monitoring theory, the CSE is caused by high conflict on incongruent trials leading to a temporary up-regulation of attentional control. The heightened top-down control enhances conflict resolution in subsequent trials, resulting in a smaller congruency effect.

Associative accounts of the CSE, on the other hand, are based on bottom-up processes. According to the feature integration account (Frings et al., 2020; Hommel, 2004), the co-occurrence of specific stimulus and response features creates an integrated episodic representation. A subsequent activation of one of these features automatically activates other associated features. Thus, the CSE occurs when there is partial (but not complete) overlap between the event file from the previous trial and the stimulus and response features on the current trial. That is, when the features of the event file partially repeat from the previous trial, the bound features are automatically activated, which then compete with the current features and lead to slower responses (e.g., the word “RED” printed in blue ink presented after the word “RED” printed in red ink). In contrast, complete repetition or no repetition of features leads to faster RTs because there are no competing features. Mayr and colleagues (2003) observed that in a task with two stimulus–response (S-R) pairs, cC trials and iI trials are always either complete feature repetitions or complete alternations, resulting in faster responses (e.g., In an arrow-flanker task, cC trials could be represented as >  >  > following >  >  > or, <  <  < , while iI trials could be <  >  < following <  >  < or, >  < >). This bottom-up account explains the CSE without attentional modulation.

In line with this bottom-up account, some studies show that the CSE is absent or reduced after controlling for feature repetition (Chen & Melara, 2009; Mayr et al., 2003; Nieuwenhuis et al., 2006). However, several studies that controlled for bottom-up confounds by expanding the stimulus set or analyzing trials without feature repetition confirmed the presence of the CSE (Akçay & Hazeltine, 2007, 2011; Duthoo & Notebaert, 2012; Kim & Cho, 2014; Notebaert & Verguts, 2006).

Another bottom-up explanation for the CSE is contingency learning (Schmidt & De Houwer, 2011; Schmidt et al., 2007). Contingency learning refers to the bottom-up processes by which participants learn predictive relationships between stimuli and responses. In conflict tasks, participants may learn the associations between specific stimuli and the likelihood of certain responses. For example, in a Stroop task, the word “RED” printed in red ink might be presented more frequently than “RED” printed in blue or green ink. Over time, participants learn that certain stimuli are more likely to be followed by certain types of trials. Consequently, they respond faster and more accurately in high contingency trials. Because the contingency effect is larger following high-contingency trials (Schmidt et al., 2007), the Stroop effect between congruent (high contingency) and incongruent (low contingency) trials can be increased following a congruent trial compared to an incongruent one. Thus, if a conflict task includes contingency learning confounds, it may induce a sequential contingency effect that affects the magnitude of the CSE.

### Confound minimized congruency sequence effect

To examine the presence of the CSE without these bottom-up confounds, researchers developed a confound-minimized procedure that involves splitting a four alternative forced choice (4-AFC) task into two separate 2-AFC tasks (Kim & Cho, 2014; Schmidt & Weissman, 2014). For example, Kim and Cho (2014) combined two Simon tasks. To avoid feature repetition/integration, they used two different stimulus sets and two different response sets (index and middle finger, ring and small finger) for each task. This also controls contingency learning because it allows for congruent and incongruent trials to be presented an equal number of times. Using this and similar confound-minimized procedures, many studies have observed CSE without feature integration and contingency learning confounds (Grant et al., 2020; Schmidt & Weissman, 2014; Weissman et al., 2020). These results, significant CSE without bottom-up confounds, support top-down attentional modulation accounts such as the conflict monitoring theory (Botvinick et al., 2001, 2004).

### Congruency sequence effect: domain-general vs domain-specific

In addition to the roles top-down or bottom-up mechanisms play in the CSE, another controversy in the literature is how domain-general these mechanisms are. This question is typically addressed with dual-task designs where the effects of the congruency of a trial in one task are assessed in the other task. Many studies support the hypothesis that control is domain-specific to a task by indicating that CSEs do not cross task boundaries (Akçay & Hazeltine, 2008, 2011; Egner et al., 2007; Funes et al., 2010a; Hazeltine et al., 2011). In line with this idea, recent dual-task studies have found within-task CSEs but no significant between-task CSEs (Aczel et al., 2021; Li et al., 2015; Torres-Quesada et al., 2013; Weissman, 2020; Yang et al., 2022). However, two studies reported domain-general CSE using the Stroop and flanker tasks (Freitas et al., 2007) or CSE across different domains (e.g., verbal and perceptual; Kan et al., 2013), partially supporting the presence of domain-general top-down control (Aczel et al., 2021). Thus, it remains unclear whether and to what extent the CSE is domain-general or domain-specific. This meta-analysis on dual-task CSEs addresses the question of domain-general vs. domain-specific control by examining both with and between tasks CSEs.

A third question examines whether the congruency effect is correlated with the CSE. As previously discussed, the congruency effect reflects conflict within a trial, while the CSE captures trial-to-trial adaptive control. It is unclear what the relationship is between these situations requiring control. Still, the larger the congruency effect is, the larger the potential is for it to change across trials. Only two studies (Colzato et al., 2016; Weissman et al., 2014) have reported no correlation between the congruency effect and CSE, suggesting that further empirical evidence is critical. The relationship between the congruency effect and the CSE has received less attention than other effects, such as top-down/bottom-up and global/local effects described above. This meta-analysis aims to address this deficiency.

### The current meta-analysis

The current meta-analysis seeks to address four research questions. First, to measure the contribution of the top-down and bottom-up factors in CSE, we calculated the magnitude of CSE from studies including bottom-up confounds and studies that used confound minimized paradigm. Second, we examine the within-task CSE and between-task CSE in dual task studies to investigate the domain-general vs. conflict-specific mechanisms for the CSE. Third, we investigated the relationship between the congruency effect and CSE. Finally, we investigated which experimental factors influence the size of CSE (e.g., Task types, Number of S-R mappings).

## Method

### Literature search

All studies were identified through a search of the web databases: PubMed and Google Scholar and using the keywords: *congruency sequence effect*, *conflict adaptation*, *Gratton effect*, *sequential modulation effect*, and *sequential effect*. The search was restricted to journal articles published in international journals and dissertations written in English. The below inclusion criteria were applied during the literature search. This literature search collected papers published from 1992 through December 2023.

### Inclusion criteria and classification

The following inclusion criteria were applied in the selection of studies:

1. The experiment recruited healthy young adult (age range: 18–39 years) participants. Studies that only employed other age groups (e.g., children, adolescents, older adults), or clinical populations (e.g., Depression, ADHD, Schizophrenia, etc.) were excluded, as these populations may show a smaller CSE or different patterns (for a review of CSE studies employed clinical populations, see Clayson et al., 2024).
2. The experiment measured a cognitive version of CSE from a standard conflict task (Stroop, flanker, Simon, spatial Stroop, prime probe, and temporal flanker task). Experiments that employed affective stimuli or conducted mood induction prior to or between the tasks were excluded.
3. The experiment provided RTs and *F*-statistics for the CSE. In the four cases where the statistical report was not included in the publication, we contacted authors to obtain the necessary statistics. We requested this information from four authors and received three replies.
4. The experiment employed a 50/50 proportion for the congruent trials and the incongruent trials. In conflict tasks, the congruency effects are larger when the proportion of congruent to incongruent trials is higher, which is referred to as the proportion congruent effect (Lowe & Mitterer, 1982). Based on conflict monitoring theory (Botvinick et al., 2001), the increased amount of conflict could increase CSE when incongruent trials are infrequent. Given that the proportion of trial congruency and the magnitude of CSE may interact, we included only studies that used an equal number of congruent and incongruent trials (26 studies excluded).
5. The study used manual key responses. Studies used other response devices (e.g., voice, mouse trajectory, foot pedals) were excluded to ensure differences in overall response time did not affect the results (7 studies excluded).

**Fig. 2:**
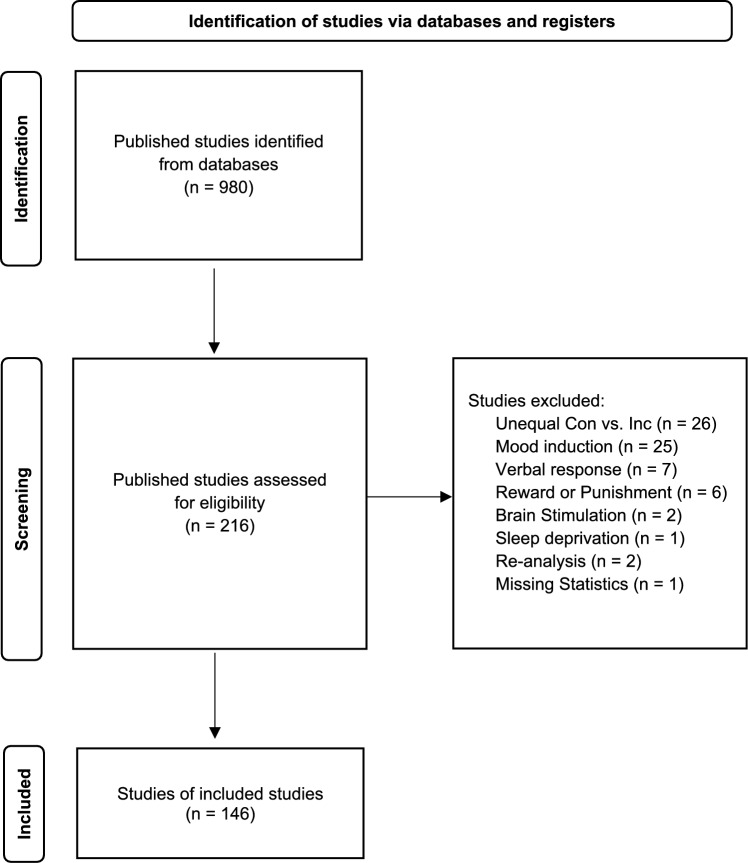
PRISMA flow diagram for study selection and evaluation in the meta-analysis. We collected studies that recruited young healthy adults that measured a cognitive version of the Congruency Sequence Effect (CSE) using a 50/50 proportion of congruent/incongruent conditions with key responses. After applying the inclusion criteria, the literature search resulted in 146 published studies, encompassing 311 independent experiments

After applying the inclusion criteria, the literature search yielded a total of 146 published studies, comprising a total of 311 independent experiments. Figure 2 shows the PRISMA flow diagram of literature search. Next, each experiment was systematically categorized using the following coding variables: tasks (dual task, flanker, prime probe,^1^ Simon, spatial Stroop, and Stroop), stimulus type (arrow, color, letter, number, words, shapes, etc.), number of S-R mappings, inter-stimulus interval, sample size, mean RTs for congruency effect and CSE, utilization of the confound minimized design, and single or combined task (e.g., combined flanker and Simon task).

### Effect size calculation

The aim of the meta-analysis was to measure the mean effect sizes for each category of CSE. To do so, we followed the procedure outlined in Borenstein and colleagues (2009) and calculated the effect size of the CSE for each study by transforming the *F*-value from the interaction between previous and current congruency to Hedges’ *g* (Hedges, 1982) as follows.

First, we calculated partial *eta* squared using this equation:

$$\eta {2}_{P}=\frac{F}{F+N-1}$$

The partial *eta* squared was then transformed into Cohen’s *d* (Cohen, 1988) using this equation:

$$Cohen{\prime}s d=\sqrt{\frac{(N-1)}{N}}\times \frac{\eta {2}_{P}}{(1-\eta {2}_{P})}$$

Then, the Cohen’s *d* was transformed into Hedges’ *g* to adjust for small sample bias using this equation:

$${g}_{z}={d}_{z }\times (1-\frac{3}{4\times (N-1)-1})$$

All reported *F*-values were transformed to Hedges’ *g*. If the CSE was not significant and the *F*-value was not reported, the effect size was entered as zero. With this procedure, a total of 28 experiments were interpreted as having a zero-effect due to nonsignificant CSE and missing *F*-value. This transformation allows us to be conservative in our estimate of the average effect size and reduce the risk of Type 1 error.

### Meta-analysis and meta-regression

We performed a series of meta-analyses to calculate the mean effect size for each category (all studies, flanker, dual task, prime probe, Simon, Stroop, between-tasks, within-task, confound minimized, feature repetition included, 2-AFC, 3-AFC, 4-AFC, and 6-AFC). Then, meta-regression analyses were conducted to identify the moderator effect of experimental factors on the CSE (task types, within vs. between tasks, repetition included, confound-minimized, number of trials, number of S-R mappings). All analyses used random-effect models, which assume the mean effect size is weighted by each experiment’s variances. The analyses were conducted using the *metafor* package in R (Viechtbauer, 2010).

To correct for publication bias, we performed an Egger’s test (Egger et al., 1997) and conducted trim-and-fill analysis (Duval & Tweedie, 2000) to estimate the effect size without bias. The threshold of statistical significance for the mean effect size analysis, as well as for the meta-regression, was set to α = 0.05.

## Results

In total, 146 studies were included, comprising 311 independent experiments. Across all studies, the mean effect size of CSE was significantly different from zero, *g* = 1.11 (CI 95%: 1.02–1.21). According to Cohen (1992), a Hedges’ *g* of ≥ 0.2 is regarded as a small effect, ≥ 0.5 is a medium effect, and ≥ 0.8 is regarded as a large effect. The heterogeneity test revealed significant heterogeneity between studies [*Q*(310) = 3,132.88, *p* < 0.001, *I*^2^ = 91.85%], warranting moderator analysis.

**Fig. 3:**
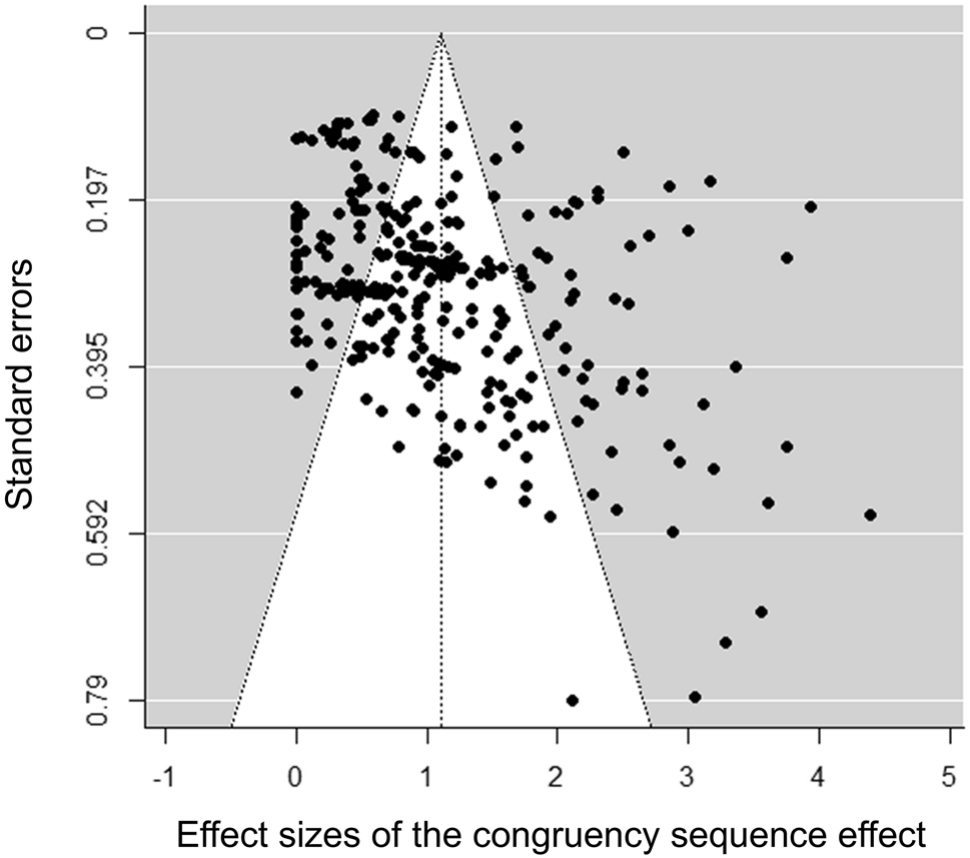
Funnel plot for effect sizes from all studies. The X-axis represents the magnitudes of the CSEs in each study, while the Y-axis represents the standard errors. Note that 28 effect sizes were set to zero when the authors reported a nonsignificant effect without accompanying *F*-values

The funnel plot of the included studies is shown in Fig. 3. There was significant evidence for publication bias in the Egger’s test for funnel plot asymmetry, *z* = 8.16, *p* < 0.001. Asymmetry in the funnel plot can suggest publication bias, where smaller studies with non-significant or unfavorable results are less likely to be published (Sterne & Egger, 2001). We used the trim-and-fill method to assess and correct for publication bias. The analysis did not identify any missing studies, indicating that the observed effect sizes remain consistent. The number of studies for each category and their mean effect size are presented in Table 1.

**Table 1:** Meta-analysis and Meta-regression results

	N	Hedge’s *g*	Lower limits of 95% CI	Upper limits of 95% CI	*Q*
Overall	311	1.11	1.02	1.21	
Task Type					13.63***
Stroop	44	0.86	0.66	1.06	
Flanker	95	1.07	0.89	1.25	
Simon	66	1.46	1.21	1.72	
Prime probe	75	1.13	0.97	1.29	
Dual Task (local vs global)	27	0.79	0.54	1.04	32.26***
Within Task	11	1.54	0.95	2.13	
Between Tasks	19	0.28	0.15	0.40	
Repetition-Included	55	1.28	1.09	1.48	9.18***
Confound-Minimized	99	0.95	0.82	1.07	5.23*
Total Number of Trials	209				5.10*
No. of S-R mappings					30.03***
2-AFC	117	1.44	1.26	1.62	
3-AFC	15	1.07	0.60	1.53	
4-AFC	173	0.91	0.80	1.01	
6-AFC	6	0.64	0.15	1.12	
Inter-Stimulus-Interval	217				1.14

Note. *N* = number of studies, *Q* is for moderator effects

^*^ *p* < .05, ** *p* < .01, ***p < .001

Meta-regression analyses were conducted to identify the moderating effect of each factor on the CSE. The first moderator was bottom-up confounds (both feature repetition and contingency learning^2^). The feature repetition significantly varies the magnitude of the CSE [R^2^ = 2.5, Q(1) = 9.18, *p* < 0.001], as the mean effect size for repetition-included studies, *g* = 1.28, *p* < 0.001, and for repetition-excluded, *g* = 1.08, *p* < 0.001, were significantly different. Most importantly, we analyzed the magnitude of CSEs in confound minimized design, which controlled for both feature repetition and contingency learning. Although the effect size is smaller than that observed in studies with confound [R^2^ = 1.35, Q(1) = 5.23, *p* < 0.05], a large weighted effect size from all confound-minimized studies, *g* = 0.95, *p* < 0.001 provides strong evidence that the CSE is robust in confound-minimized design.

To investigate whether CSE acts locally (task-specific level) or globally (domain-general control), we examined effect sizes for within-task effects (e.g., task set repeated) versus between-task effects (e.g., task set changed) within dual-task procedures. The mean effect size for the combined task was *g* = 0.76, *p* < 0.001. The within-task CSE, *g* = 1.54, *p* < 0.001, was larger than the between-task CSE, *g* = 0.27, *p* < 0.001; [*Q*(1) = 32.26, *p* < 0.001]. This result supports theories claiming that the CSE acts strongly at a task-specific level, although it also indicates there is a global effect as well.

We performed a meta-regression analysis of the different types of tasks (Stroop, flanker, Simon, and prime probe). Task type significantly explained 9% of the variance [R^2^ = 3.23, *Q*(3) = 13.63, *p* < 0.001]. The mean effect size (Hedges’ *g*) for flanker, Simon, Stroop, and prime-probe tasks was 1.07, 1.46, 0.86, and 1.13, respectively (*p* < 0.001, for all effect sizes). The largest CSE observed in the Simon task might be due to the fact that most studies using this task did not minimize confounds. Of the 66 studies on the Simon task, 55 included bottom-up confounds, while only 15 were confound-minimized. In contrast, for the prime-probe task, 45 studies were confound-minimized, and 30 studies had bottom-up confounds. This is supported by meta-regression results indicating a significant difference in CSE between confound minimized and non-minimized design in the Simon Task [R^2^ = 14.85, *Q*(1) = 10.85, *p* < 0.001]. However, there was no significant differences for confound minimized in other tasks [all *ps* < 0.05]. In addition, a meta-regression, conducted only for the confound minimized studies, showed that task type did not significantly modulate the size of CSE [*Q*(4) = 7.22, *p* = 0.12].

Additionally, we investigated experimental design factors that influence the size of CSE. The number of trials significantly moderated the CSE, [*Q*(1) = 5.10, *p* = 0.024], with a significant positive correlation between total number of trials and CSE, [*r*(207) = 0.16, *p* = 0.025]. The inter-stimulus interval (ISI) did not alter the magnitude of CSE, [*Q*(1) = 1.14, *p* = 0.286]. The data set contained the following number of S-R mappings: 2-AFC (13 Stroop, 49 flanker, 33 Simon, 8 prime probe, 12 dual task, 2 spatial Stroop), 3-AFC (6 Stroop, 9 flanker), 4-AFC (23 Stroop, 35 flanker, 33 Simon, 65 prime probe, 15 dual task, 2 spatial Stroop), and 6-AFC (2 Stroop, 2 flanker, 2 prime probe). The number of S-R mappings was significantly related to effect size, [*Q*(3) = 30.03, *p* < 0.001], with a decreasing CSE as the number of S-R mappings increased: The mean effect size (*g*) for CSE in 2-AFC, 3-AFC, 4-AFC, and 6-AFC was 1.44, 1.07, 0.91, and 0.64, respectively (*p* < 0.001, for all analyses, *r*(309) = −0.299, *p* < 0.001). The results showed that the CSE decreases as the number of S-R mappings increases. However, as discussed previously, 2-AFC tasks have bottom up confounds, which likely drives larger CSEs. In the meta-regression analysis of studies with minimized confounds, ISI did not significantly moderate the size of CSE [*Q*(15) = 22.92, *p* = 0.09], while the number of trials significantly affected the magnitude [*Q*(29) = 45.65, *p* = 0.03]. The number of S-R mappings could not be examined because the majority of confound minimized studies used a 4-AFC design.

Lastly, we tested the correlation between the congruency effect and CSE. In this analysis, only experiments that reported mean RTs for the congruency effect and CSE were included (203 experiments). The mean congruency score was 54.45 ms (*SD* = 39.60 ms) and the mean CSE was 29.19 ms (*SD* = 24.05 ms). The correlation was not significant, [*r*(202) = −0.08, *p* = 0.21]. In addition, we performed meta-regression analysis by treating the congruency score as a continuous variable, and the results showed that the congruency effect was not a significant moderator of CSE, [*Q*(134) = 98.28, *p* = 0.99].

## Discussion

In summary, the overall effect size of the CSE was 1.11 (Hedges’ *g*). There was a large and significant CSE across tasks and procedures. The funnel plot analysis suggested the possibility of publication bias but after correcting for this possibility, the overall effect size remained significant. We found significant heterogeneity between studies, warranting further moderator analyses.

First, we calculated the effect size of the CSE from studies including bottom-up confounds (viz., feature binding and contingency learning) and studies that controlled for these confounds (confound minimized procedures). Results showed that bottom-up confounds drive some of the CSE, which was larger for designs including confounds (Hedges’ *g* = 1.28). However, CSE remained robust and large in confound-minimized designs (Hedges’ *g* = 0.95). This result confirms the presence of the CSE in the absence of feature binding or contingency learning, which likely reflects top-down attentional control processes produce the CSE. Egner (2014) proposed an integrated perspective in which top-down control and bottom-up priming work together complementarily, rather than in opposition, toward a common goal. Recent descriptions of the binding account (Frings et al., 2020; Schiltenwolf et al., 2024) suggest that task rules and context features (e.g., congruency) may be part of the bound representation, and repetition of contextual features in a subsequent trial may facilitate the retrieval of these task rules (e.g., encountering an incongruent trial after an incongruent one). Thus, the binding processes may involve response-independent control over the CSE, which provides more integrated perspective on top-down versus bottom-up influences.

Second, previous studies reported conflicting evidence about whether the CSE is the result of a domain-general or task-specific process (Akçay & Hazeltine, 2008, 2011; Egner et al., 2007; Freitas et al., 2007; Funes et al., 2010a; Hazeltine et al., 2011; Kan et al., 2013; Rünger et al., 2010). To investigate this, we compared the size of CSE within a task (which would include both specific and general processes) and between tasks (where only general processes would be at play). Robust and significant CSE within a task (Hedges’ *g* = 1.54) showed that CSE is more driven by a domain-specific control. In line with this result, recent studies have found a within-task CSE but failed to observe a significant CSE between tasks CSE (Aczel et al., 2021; Li et al., 2015; Torres-Quesada et al., 2013; Weissman, 2020; Yang et al., 2022). However, we also found a small but significant CSE between tasks (Hedge’s *g* = 0.27). These results suggest that, even though the mechanisms for the CSE are predominantly local, some degree of CSE may be driven by domain-general control. The observed domain-general CSE may reflect control processes at higher level of the task representation.

For the domain-specific CSE, it remains an open question what level of task representation determines the boundaries of domain-specific control process. Our laboratories have proposed that the task set (i.e., representation of the current task) determines the boundaries of control (e.g., CSE is not observed between different task sets), rather than single stimulus or response representations (Hazeltine et al., 2011; Schumacher & Hazeltine, 2016). Using a cross-modal prime-probe task, Grant and colleagues (2020) found that the CSE does not carry over when task sets changed, indicating that CSE is specific to the context of the task set. Additionally, Lim and Cho (2018, 2021) reported that dual-task CSEs were only significant when the two tasks had the same response rule, suggesting that the response mode may modulate CSE. Finally, Yang and colleagues (2021) suggested that the similarity of conflicts may modulate the transfer of CSE across tasks. They manipulated the task similarity in the Simon and spatial Stroop task by varying the degree of feature overlap, finding that greater similarity between incongruent trials in tasks resulted in a larger between-task CSE. More recently, Zhu and colleagues (2024) conducted a meta-analysis on cross task-CSEs, observing a significant effect size for between-task CSE. They found a negative relationship between task-dissimilarity and size of between-task CSE. These findings provide important future questions regarding the task boundaries under which CSEs transfer across tasks.

Third, we investigated the experimental factors that may moderate the size of CSE (c.f., Braem et al., 2019). We measured the magnitude of CSE in different tasks. The results revealed that task type significantly affects the size of the CSE. The Simon task (Hedges’ *g* = 1.46) at least nominally evoked the largest effect size, followed by the prime probe, the flanker, and the Stroop task (Hedges’ *g* = 1.13, 1.07 and 0.86). This falls in line with previous research showing that the CSE in Stroop task is reliable, but the effect size is relatively small (Whitehead et al., 2019). Studies using the Simon task produced the largest CSEs, but this is likely caused by the effect of bottom-up confounds rather than the task itself because the majority of Simon studies included bottom-up confounds by employing a 2-AFC paradigm. When we analyzed the task type effect within confound-minimized studies (Hedges’ g = 0.95). There was no significant difference in effect sizes between tasks, indicating that all the tasks elicit a stable and similar level of CSE when confounds are minimized.

Our results indicate that the prime-probe tasks produced a large CSE (Hedges’ *g* = 1.13). This finding aligns with other studies that have reported large CSEs in tasks where the distractor is presented before the target, such as the prime-probe task and temporal flanker (Hazeltine et al., 2011; Schmidt & Weissman, 2014). Interestingly, prime probe tasks consistently produce larger CSEs than tasks that present the target and distractor at the same time (Bombeke et al., 2017; Weissman et al., 2015). The prime-probe tasks are particularly well-suited for the confound minimized experiments and have flexibility and control over experimental factors. Researchers can manipulate the type of prime and probe, the number of S-R mappings, duration of stimulus presentation and the cue-stimulus intervals (CSI). However, one limitation is that the congruency effect disappears with a long CSI condition (e.g., 1000 ms; Weissman et al., 2015). Therefore, tasks that can separate the distractors from targets, such as the prime-probe task, are useful for minimizing bottom-up confounds and addressing future questions related to the CSE.

In addition, this meta-analysis demonstrates that the magnitude of the CSE significantly decreases as the number of stimulus–response pairs in a task increases. The result aligns with findings from a numerical flanker task, where the size of CSE decreased as the number of unique stimuli increased (Blais & Verguts, 2012). Because studies employing 2-AFC tasks often contain feature repetition effects, they tend to show larger CSEs compared to 4-AFC or 6-AFC tasks. When the number of trials is equal for both 2-AFC and 4-AFC tasks, stimulus repetition occurs more frequently in 2-AFC tasks (e.g., in a 2-AFC flanker task, 'HHHHH' might appear 60 times in 240 trials, whereas in a 4-AFC flanker task, it might appear only 30 times in 240 trials). As a result, smaller CSEs are associated with studies that use more S-R pairs. Additionally, increasing the number of S-R pairs can impact the efficiency of top-down control, likely due to higher cognitive demands and task difficulty. Even when specific stimulus features are not presented in current trials, they remain part of the task set, requiring participants to maintain a more complex task representation. This increased complexity may heighten conflict and task difficulty. Supporting this idea, Soutschek and colleagues (2013) found that the CSE disappeared under high working memory load conditions. Furthermore, in both Stroop and flanker tasks, the increased number of S-R mappings is associated with slower RTs, larger congruency effect, and prolonged incongruent-related brain activity, which may influence the efficiency of top-down control (Donohue et al., 2016). van Steenbergen and colleagues (2015) suggested that high task difficulty can diminish the CSE, although the overall relationship appears to be U-shaped. We are unable to analyze this question for confound minimized studies because of the limited number of studies that used more than 4-AFC mappings. Future research using confound minimized designs may investigate the relationship between task complexity and the CSE in more detail.

The number of experimental trials showed a weak positive correlation with the size of the CSE (*r* = 0.16). However, inter-stimulus interval (ISI) did not significantly affect the size of the CSE. Note, however, that in our dataset most of the experiments used an ISI of around 1000 ms (M:1217.74 ms, SD: 667.83 ms). Some studies have tested the time-course of the CSE at different ranges, specifically, 50 versus 200 ms (Notebaert et al., 2006), 1500 versus 6000 ms (Wühr & Ansorge, 2005). Results from these two studies indicate that the CSEs are larger at short ISI and decay with increasing ISI (Egner et al., 2010). Our results suggest the CSE is robust for small variations of ISI centering around 1000 ms, but more research is necessary to determine the effect on the CSE of very short or very long ISIs. Additionally, the congruency sequence effect in the prime-probe task can be influenced by the CSI. In our dataset, some studies used a CSI of 133 ms (e.g., Hazeltine et al., 2011), while others used 1000 ms (e.g., Weissman et al., 2020). However, due to the limited number of experiments, we grouped all prime-probe tasks as a single variable in our analysis.

Finally, we examined the relationship between the congruency effect and the CSE. As earlier studies have reported (Colzato et al., 2016; Weissman et al., 2014), the magnitude of CSE does not correlate with the size of the congruency effect. These results may suggest that the response selection within a trial and the flexible attentional modulation between trials might be derived from distinct control mechanisms. It is also plausible that efficient conflict resolution (e.g., smaller Stroop effect) may not modulate the degree of subsequent behavioral adaptation (e.g., CSE), rather only the conflict detection itself drives the subsequent attentional modulation. Even though the CSE is a modulation of the congruency effect across trials, there was no systematic relationship between the two effects. Note that we measured the between-study correlation, examining whether studies with a larger congruency effect tend to yield a larger CSE. However, the most effective way to test this relationship would be through a meta-analysis of within-study correlations. Unfortunately, we were unable to perform this analysis because very few studies reported the correlation between congruency score and CSE. An interesting future question is how the control in a trial and flexible control across the trials interact.

A remaining question is whether and how the proportion of congruency affects the magnitude of the CSE. Previous studies have shown that a high proportion of incongruent trials reduces congruency effects, while a low proportion of conflict results in increased congruency effects (Bugg & Crump, 2012; Carter et al., 2000; Casey et al., 2000; Gratton et al., 1992). Frequent presentation of incongruent stimuli may activate more sustained, anticipatory control processing (Braver, 2012; Kane & Engle, 2003), which may interfere with the transient control adjustments involved in the CSE (Duthoo et al., 2014). Purmann and colleagues (2009) reported that the CSE decreased in a high frequency of incongruent trials. However, several studies have indicated a dissociation between the proportion congruency effect and the CSE, suggesting that each effect arises from different control mechanisms and can occur independently, with little to no correlation between them (Funes et al., 2010b; Mayr & Awh, 2009; Torres-Quesada et al., 2013; Torres-Quesada et al. (2014). Among the studies excluded from our literature search, the proportion of incongruent trials varied (e.g., 75%, 70%, 65%, or 62.5%), and the size of the CSE may also be confounded by experimental factors such as contingency and the number of S-R mappings. Consequently, we could not address this question as it was beyond the scope of the current meta-analysis. However, a multi-level meta-analysis that systematically reviews the literature while accounting for these factors could be a possible direction for future research.

### Individual differences in CSE

The current study has limitations associated with our meta-analytic approach. One is the inability to investigate individual differences within experiments. Previous studies have found several variables that at least sometimes affect the magnitude of the CSE at the individual level, such as working memory capacity, motivation, intelligence, and bilingualism. Using the Simon task, Weldon and colleagues (2013) found that individuals with high working memory capacity displayed less CSE after incongruent trials compared to those with low working memory capacity. However, several studies using the Stroop and flanker task found that the working memory capacity does not interact with CSE (Keye et al., 2013; Meier & Kane, 2013; Unsworth et al., 2012; Wiemers & Redick, 2018). For motivation, studies found that participants with high motivation produced larger CSEs than those with low motivation scores (Zhao et al., 2018). For intelligence, Liu and colleagues (2016) found that the high IQ participants had better performance for CSE than the average IQ group. In addition, studies investigating bilingual subjects have found inconsistent results. Grundy and colleagues (2017) report that CSE is smaller for bilingual than monolingual subjects. In contrast, Paap and colleagues (2019) and Goldsmith and Morton, (2018) observed no differences in CSE between bilingual and monolingual subjects. Unfortunately, we were not able to consider these individual differences factors with this meta-analysis because the current state of the literature does not allow for these factors to be investigated in a between-study context.

## Conclusions

These meta-analyses investigated several theoretical controversies in literature. We found that the CSE was larger for studies that included bottom-up effects than studies that controlled for these confounds. This suggests that the bottom-up effects of feature binding and contingency learning play a role in the CSE (c.f., Hommel, 2004; Mayr et al., 2003; Schmidt & De Houwer, 2011). However, after removing these confounds, the CSE is robust and significant, suggesting that top-down mechanisms also play a role in the CSE. Similarly, the CSE was larger within tasks than between tasks. This suggests that the mechanisms for the CSE act at a local (task) level (c.f., Akçay & Hazeltine, 2011; Egner et al., 2007; Hazeltine et al., 2011; Schumacher et al., 2011). However, there was a robust and significant CSE across tasks, suggesting that global mechanisms also play a role (c.f. Freitas et al., 2007; Kan et al., 2013). Additional results suggest that the mechanisms for the congruency effect may not be the same as those for the CSE. Finally, given the relatively large CSE effect size for the prime probe and temporal flanker tasks and the simple way these tasks can be modified to minimize bottom-up confounds, our results suggest these tasks may be especially useful for answering additional questions about the CSE in future research.

## Supplementary Information

Below is the link to the electronic supplementary material.

Supplementary file1 (DOCX 28 KB)

## Data Availability

No datasets were generated or analysed during the current study.

## Footnotes

1 Given the similarity of the designs and the small number of temporal flanker studies, temporal flanker and prime probe were combined in the analysis.

2 Distinguishing between feature repetition and contingency learning within the context of the Congruency Sequence Effect (CSE) is challenging because they frequently co-occur. In our meta-analysis, we examined studies that included repetition effects and minimized both confounds. However, we did not quantify the magnitude of the CSE for studies that controlled for feature repetition but still faced contingency learning confounds.
