# Supplementary material for: Meta-analytic evidence for the complex mechanisms underlying congruency sequence effect
Source: Psychol Res. 2025 Mar 6;89(2):63. doi: 10.1007/s00426-025-02093-5 (PMC11885397; doi:10.1007/s00426-025-02093-5)
Supplement: Supplementary file 1 — Supplementary file1 (DOCX 28 KB) [file 426_2025_2093_MOESM1_ESM.docx]

**References list of Studies Included in the Meta-Analysis.**

Akçay, Ç., & Hazeltine, E. (2007). Conflict monitoring and feature overlap: Two sources of sequential modulations. *Psychonomic bulletin & review, 14*(4), 742-748.

Akçay, Ç., & Hazeltine, E. (2008). Conflict adaptation depends on task structure. Journal of Experimental Psychology: *Human perception and performance, 34*(4), 958.

Akçay, Ç., & Hazeltine, E. (2011). Domain-specific conflict adaptation without feature repetitions. *Psychonomic Bulletin & Review, 18*(3), 505-511.

Bausenhart, K. M., Ulrich, R., & Miller, J. (2021). Effects of conflict trial proportion: A comparison of the Eriksen and Simon tasks. *Attention, Perception, & Psychophysics, 83*(2), 810-836.

Bissett, P. G., Grant, L. D., & Weissman, D. H. (2017). Resisting distraction and response inhibition trigger similar enhancements of future performance. *Acta Psychologica, 180*, 40-51.

Blais, C., & Verguts, T. (2012). Increasing set size breaks down sequential congruency: Evidence for an associative locus of cognitive control. *Acta Psychologica, 141*(2), 133-139.

Bombeke, K., Langford, Z. D., Notebaert, W., & Boehler, C. N. (2017). The role of temporal predictability for early attentional adjustments after conflict. *PloS one, 12*(4), e0175694.

Braem, S., Hickey, C., Duthoo, W., & Notebaert, W. (2014). Reward determines the context-sensitivity of cognitive control. Journal of Experimental Psychology: *Human perception and performance, 40*(5), 1769.

Braem, S., Verguts, T., & Notebaert, W. (2011). Conflict adaptation by means of associative learning. Journal of Experimental Psychology: *Human Perception and Performance, 37*(5), 1662.

Braem, S., Verguts, T., Roggeman, C., & Notebaert, W. (2012). Reward modulates adaptations to conflict. *Cognition, 125*(2), 324-332.

Brosowsky, N. P., & Crump, M. J. (2018). Memory-guided selective attention: Single experiences with conflict have long-lasting effects on cognitive control*. Journal of Experimental Psychology: General, 147*(8), 1134.

Bugg, J. M. (2008). Opposing influences on conflict-driven adaptation in the Eriksen flanker task. *Memory & Cognition, 36*(7), 1217-1227.

Burle, B., Allain, S., Vidal, F., & Hasbroucq, T. (2005). Sequential compatibility effects and cognitive control: does conflict really matter?. *Journal of Experimental Psychology: Human Perception and Performance, 31*(4), 831.

Cho, R. Y., Orr, J. M., Cohen, J. D., & Carter, C. S. (2009). Generalized signaling for control: Evidence from postconflict and posterror performance adjustments. Journal of Experimental Psychology: *Human Perception and Performance, 35*(4), 1161.

Clayson, P. E., & Larson, M. J. (2011). Conflict adaptation and sequential trial effects: Support for the conflict monitoring theory. *Neuropsychologia, 49*(7), 1953-1961.

Clayson, P. E., & Larson, M. J. (2012). Cognitive performance and electrophysiological indices of cognitive control: A validation study of conflict adaptation. *Psychophysiology, 49*(5), 627-637.

Clayson, P. E., & Larson, M. J. (2013). Psychometric properties of conflict monitoring and conflict adaptation indices: Response time and conflict N 2 event‐related potentials. *Psychophysiology, 50*(12), 1209-1219.

Colzato, L. S., Sellaro, R., Hulka, L. M., Quednow, B. B., & Hommel, B. (2014). Cognitive control predicted by color vision, and vice versa. *Neuropsychologia, 62*, 55-59.

Correa, Á., Rao, A., & Nobre, A. C. (2009). Anticipating conflict facilitates controlled stimulus-response selection. *Journal of cognitive neuroscience, 21*(8), 1461-1472.

Davelaar, E. J. (2013). When the ignored gets bound: Sequential effects in the flanker task. *Frontiers in psychology, 3*, 552.

de Galan, M., Sellaro, R., Colzato, L. S., & Hommel, B. (2014). Conflict adaptation is predicted by the cognitive, but not the affective alexithymia dimension. *Frontiers in Psychology, 5*, 768.

Desender, K., Van Opstal, F., & Van den Bussche, E. (2014). Feeling the conflict: The crucial role of conflict experience in adaptation. *Psychological science, 25*(3), 675-683.

Dignath, D., Kiesel, A., & Eder, A. B. (2015). Flexible conflict management: conflict avoidance and conflict adjustment in reactive cognitive control. *Journal of Experimental Psychology: Learning, Memory, and Cognition, 41*(4), 975.

Dignath, D., Kiesel, A., Schiltenwolf, M., & Hazeltine, E. (2021). Multiple Routes to Control in the Prime-Target Task: Congruence Sequence Effects Emerge Due to Modulation of Irrelevant Prime Activity and Utilization of Temporal Order Information. *Journal of Cognition, 4*(1).

Duthoo, W., Abrahamse, E. L., Braem, S., & Notebaert, W. (2014). Going, going, gone? Proactive control prevents the congruency sequence effect from rapid decay. *Psychological Research, 78*(4), 483-493.

Duthoo, W., Abrahamse, E. L., Braem, S., Boehler, C. N., & Notebaert, W. (2014). The congruency sequence effect 3.0: a critical test of conflict adaptation. *PloS one, 9*(10), e110462.

Egner, T., Ely, S., & Grinband, J. (2010). Going, going, gone: characterizing the time-course of congruency sequence effects. *Frontiers in psychology, 1*, 154.

Erb, C. D., & Cavanagh, J. F. (2019). Layers of latent effects in cognitive control: An EEG investigation. *Acta Psychologica, 195*, 1-11.

Erb, C. D., McBride, A. G., & Marcovitch, S. (2019). Associative priming and conflict differentially affect two processes underlying cognitive control: Evidence from reaching behavior. *Psychonomic bulletin & review, 26*(4), 1400-1410.

Erb, C. D., Welhaf, M. S., Smeekens, B. A., Moreau, D., Kane, M. J., & Marcovitch, S. (2021). Linking the dynamics of cognitive control to individual differences in working memory capacity: Evidence from reaching behavior. *Journal of Experimental Psychology: Learning, Memory, and Cognition, 47*(9), 1383.

Feldman, J. L., & Freitas, A. L. (2018). An analysis of N2 event-related-potential correlates of sequential and response-facilitation effects in cognitive control. *Journal of Psychophysiology, 33*(2)

Fischer, R., Plessow, F., Dreisbach, G., & Goschke, T. (2015). Individual differences in the context‐dependent recruitment of cognitive control: Evidence from action versus state orientation. *Journal of Personality, 83*(5), 575-583.

Fischer, R., Plessow, F., Kunde, W., & Kiesel, A. (2010). Trial-to-trial modulations of the Simon effect in conditions of attentional limitations: Evidence from dual tasks. *Journal of Experimental Psychology: Human Perception and Performance, 36*(6), 1576.

Forster, S. E., & Cho, R. Y. (2014). Context specificity of post-error and post-conflict cognitive control adjustments. *PLoS One, 9*(3), e90281.

Freitas, A. L., & Clark, S. L. (2015). Generality and specificity in cognitive control: conflict adaptation within and across selective-attention tasks but not across selective-attention and Simon tasks. *Psychological Research, 79*(1), 143-162.

Freitas, A. L., Bahar, M., Yang, S., & Banai, R. (2007). Contextual adjustments in cognitive control across tasks. *Psychological Science, 18*(12), 1040-1043.

Fritz, J., Fischer, R., & Dreisbach, G. (2015). The influence of negative stimulus features on conflict adaption: evidence from fluency of processing. *Frontiers in psychology, 6*, 185.

Funes, M. J., Lupiáñez, J., & Humphreys, G. (2010). Analyzing the generality of conflict adaptation effects. Journal of Experimental Psychology: *Human Perception and Performance, 36*(1), 147.

Goldsmith, S. F., & Morton, J. B. (2018). Sequential congruency effects in monolingual and bilingual adults: a failure to replicate Grundy et al.(2017). *Frontiers in psychology, 9*, 2476.

Grant, L. D., & Weissman, D. H. (2017). An attentional mechanism for minimizing cross-modal distraction. *Acta psychologica, 174*, 9-16.

Grant, L. D., & Weissman, D. H. (2019). Turning distractors into targets increases the congruency sequence effect. *Acta psychologica, 192*, 31-41.

Grant, L. D., Cerpa, S. R., & Weissman, D. H. (2022). Rethinking attentional reset: Task sets determine the boundaries of adaptive control. *Quarterly Journal of Experimental Psychology, 75*(6), 1171-1185.

Grant, L. D., Cookson, S. L., & Weissman, D. H. (2020). Task sets serve as boundaries for the congruency sequence effect. *Journal of Experimental psychology: Human perception and performance, 46*(8), 798.

Gratton, G., Coles, M. G., & Donchin, E. (1992). Optimizing the use of information: strategic control of activation of responses. *Journal of Experimental Psychology: General, 121*(4), 480.

Grundy, J. G. (2022). The Specificity and Reliability of Conflict Adaptation: A Mouse-Tracking Study. *Frontiers in Psychology, 12*, 770509.

Grundy, J. G., Chung-Fat-Yim, A., Friesen, D. C., Mak, L., & Bialystok, E. (2017). Sequential congruency effects reveal differences in disengagement of attention for monolingual and bilingual young adults. *Cognition, 163*, 42-55.

Gyurkovics, M., Kovacs, M., Jaquiery, M., Palfi, B., Dechterenko, F., & Aczel, B. (2020). Registered Replication Report of Weissman, DH, Jiang, J., & Egner, T.(2014). Determinants of congruency sequence effects without learning and memory confounds. *Attention, Perception, & Psychophysics, 82*(8), 3777-3787.

Hazeltine, E., Akçay, Ç., & Mordkoff, J. T. (2011). Keeping Simon simple: Examining the relationship between sequential modulations and feature repetitions with two stimuli, two locations and two responses. *Acta Psychologica, 136*(2), 245-252.

Hazeltine, E., Lightman, E., Schwarb, H., & Schumacher, E. H. (2011). The boundaries of sequential modulations: evidence for set-level control. *Journal of Experimental Psychology: Human Perception and Performance, 37*(6), 1898.

Hemmerich, K., Narganes-Pineda, C., Marotta, A., Martín-Arévalo, E., Jiménez, L., & Lupiáñez, J. (2022). Gaze elicits social and nonsocial attentional orienting: An interplay of shared and unique conflict processing mechanisms. *Journal of Experimental Psychology: Human Perception and Performance, 48*(8), 824.

Hoppe, K., Wascher, E., & Küper, K. (2019). Feature overlap and relevance determine sequential modulations in the Simon task. *Journal of Psychophysiology, 34*(2)

Hutcheon, T. G., & Spieler, D. H. (2014). Contextual influences on the sequential congruency effect. *Psychonomic bulletin & review, 21*(1), 155-162.

Hutcheon, T. G., Spieler, D. H., & Eldar, M. (2017). Properties of context-driven control revealed through the analysis of sequential congruency effects. *Acta Psychologica, 178*, 107-113.

Iani, C., Rubichi, S., Gherri, E., & Nicoletti, R. (2009). Co-occurrence of sequential and practice effects in the Simon task: Evidence for two independent mechanisms affecting response selection. *Memory & cognition, 37*(3), 358-367.

Janczyk, M., & Leuthold, H. (2018). Effector system-specific sequential modulations of congruency effects. *Psychonomic Bulletin & Review, 25*(3), 1066-1072.

Jeong, H. J., & Cho, Y. S. (2019). Cognitive control under high threat: the effect of shock on the congruency sequence effect. *Motivation and Emotion, 43*(6), 906-916.

Jiang, J., Zhang, Q., & van Gaal, S. (2015). Conflict awareness dissociates theta-band neural dynamics of the medial frontal and lateral frontal cortex during trial-by-trial cognitive control. *Neuroimage, 116*, 102-111.

Jiménez, L., & Méndez, A. (2013). It is not what you expect: Dissociating conflict adaptation from expectancies in a Stroop task. *Journal of Experimental Psychology: Human Perception and Performance, 39*(1), 271.

Jiménez, L., & Méndez, A. (2014). Even with time, conflict adaptation is not made of expectancies. *Frontiers in Psychology, 5*, 1042.

Kalanthroff, E., Marsh, R., Hassin, R. R., & Simpson, H. B. (2020). Evidence for trial-by-trial dynamic adjustment of task control in unmedicated adults with OCD. *Behaviour Research and Therapy, 126*, 103572.

Keye, D., Wilhelm, O., Oberauer, K., & Stürmer, B. (2013). Individual differences in response conflict adaptations. *Frontiers in psychology, 4*, 947.

Keye, D., Wilhelm, O., Oberauer, K., & Van Ravenzwaaij, D. (2009). Individual differences in conflict-monitoring: Testing means and covariance hypothesis about the Simon and the Eriksen Flanker task. *Psychological Research PRPF, 73*(6), 762-776.

Kim, S., & Cho, Y. S. (2014). Congruency sequence effect without feature integration and contingency learning. *Acta psychologica, 149*, 60-68.

Kim, S., Lee, S. H., & Cho, Y. S. (2015). Control processes through the suppression of the automatic response activation triggered by task-irrelevant information in the Simon-type tasks. *Acta Psychologica, 162*, 51-61.

Kunde, W., & Wühr, P. (2006). Sequential modulations of correspondence effects across spatial dimensions and tasks. *Memory & Cognition, 34*(2), 356-367.

Langeslag, S. J., & van Steenbergen, H. (2020). Cognitive control in romantic love: the roles of infatuation and attachment in interference and adaptive cognitive control. *Cognition and Emotion, 34*(3), 596-603.

Larson, M. J., Clawson, A., Clayson, P. E., & South, M. (2012). Cognitive control and conflict adaptation similarities in children and adults. *Developmental Neuropsychology, 37*(4), 343-357.

Larson, M. J., Clayson, P. E., & Baldwin, S. A. (2012). Performance monitoring following conflict: Internal adjustments in cognitive control?. *Neuropsychologia, 50*(3), 426-433.

Larson, M. J., Clayson, P. E., Keith, C. M., Hunt, I. J., Hedges, D. W., Nielsen, B. L., & Call, V. R. (2016). Cognitive control adjustments in healthy older and younger adults: Conflict adaptation, the error-related negativity (ERN), and evidence of generalized decline with age. *Biological Psychology, 115*, 50-63.

Larson, M. J., Farrer, T. J., & Clayson, P. E. (2011). Cognitive control in mild traumatic brain injury: Conflict monitoring and conflict adaptation. *International Journal of Psychophysiology, 82*(1), 69-78.

Larson, M. J., Kaufman, D. A., & Perlstein, W. M. (2009). Neural time course of conflict adaptation effects on the Stroop task. *Neuropsychologia, 47*(3), 663-670.

Larson, M. J., LeCheminant, J. D., Carbine, K., Hill, K. R., Christenson, E., Masterson, T., & LeCheminant, R. (2015). Slow walking on a treadmill desk does not negatively affect executive abilities: an examination of cognitive control, conflict adaptation, response inhibition, and post-error slowing. *Frontiers in Psychology, 6*, 723.

Lee, J., & Cho, Y. S. (2013). Congruency sequence effect in cross-task context: evidence for dimension-specific modulation. *Acta psychologica, 144*(3), 617-627.

Li, Q., Wang, K., Nan, W., Zheng, Y., Wu, H., Wang, H., & Liu, X. (2015). Electrophysiological dynamics reveal distinct processing of stimulus‐stimulus and stimulus‐response conflicts. *Psychophysiology, 52*(4), 562-571.

Li, Z., Yang, G., Wu, H., Li, Q., Xu, H., Göschl, F., ... & Liu, X. (2021). Modality-specific neural mechanisms of cognitive control in a Stroop-like task. *Brain and Cognition, 147*, 105662.

Liepelt, R., Wenke, D., Fischer, R., & Prinz, W. (2011). Trial-to-trial sequential dependencies in a social and non-social Simon task. *Psychological Research, 75*(5), 366-375.

Lim, C. E., & Cho, Y. S. (2018). Determining the scope of control underlying the congruency sequence effect: roles of stimulus-response mapping and response mode. *Acta psychologica, 190*, 267-276.

Lim, C. E., & Cho, Y. S. (2020). Response mode modulates the congruency sequence effect in spatial conflict tasks: evidence from aimed-movement responses. *Psychological Research*, 1-22.

Lim, C. E., & Cho, Y. S. (2021). Response mode modulates the congruency sequence effect in spatial conflict tasks: Evidence from aimed-movement responses. *Psychological Research*, 85(5), 2047-2068.

Liu, P., Fei, N., Gao, S., Yang, X., Sun, J., & Qin, W. (2019). “Practice makes perfect?” white matter microstructural characteristic predicts the degree of improvement in within-trial conflict processing across two weeks. *Brain Imaging and Behavior, 13*(3), 841-851.

Liu, T., Xiao, T., & Shi, J. (2016). Fluid intelligence and neural mechanisms of conflict adaptation. *Intelligence, 57*, 48-57.

Liu, X., Liu, T., Shangguan, F., Sørensen, T. A., Liu, Q., & Shi, J. (2018). Neurodevelopment of conflict adaptation: Evidence from event-related potentials. *Developmental psychology, 54*(7), 1347.

Lorist, M. M., & Jolij, J. (2012). Trial history effects in Stroop task performance are independent of top-down control. *PloS one, 7*(6), e39802.

Mayr, U., Awh, E., & Laurey, P. (2003). Conflict adaptation effects in the absence of executive control. *Nature neuroscience, 6*(5), 450-452.

Miles, J. D., & Proctor, R. W. (2012). Correlations between spatial compatibility effects: are arrows more like locations or words?. *Psychological Research, 76*(6), 777-791.

Nieuwenhuis, S., Stins, J. F., Posthuma, D., Polderman, T. J., Boomsma, D. I., & de Geus, E. J. (2006). Accounting for sequential trial effects in the flanker task: Conflict adaptation or associative priming?. *Memory & cognition, 34*(6), 1260-1272.

Paap, K. R., & Greenberg, Z. I. (2013). There is no coherent evidence for a bilingual advantage in executive processing. *Cognitive psychology, 66*(2), 232-258.

Pastötter, B., Dreisbach, G., & Bäuml, K. H. T. (2013). Dynamic adjustments of cognitive control: oscillatory correlates of the conflict adaptation effect. *Journal of Cognitive Neuroscience, 25*(12), 2167-2178.

Plessow, F., Fischer, R., Kirschbaum, C., & Goschke, T. (2011). Inflexibly focused under stress: acute psychosocial stress increases shielding of action goals at the expense of reduced cognitive flexibility with increasing time lag to the stressor. *Journal of cognitive neuroscience, 23*(11), 3218-3227.

Puccioni, O., & Vallesi, A. (2012). High cognitive reserve is associated with a reduced age-related deficit in spatial conflict resolution. *Frontiers in human neuroscience, 6*, 327.

Puccioni, O., & Vallesi, A. (2012). Sequential congruency effects: disentangling priming and conflict adaptation. *Psychological Research, 76*(5), 591-600.

Purmann, S., & Pollmann, S. (2015). Adaptation to recent conflict in the classical color-word Stroop-task mainly involves facilitation of processing of task-relevant information. *Frontiers in human neuroscience, 9*, 88.

Questienne, L., van Dijck, J. P., & Gevers, W. (2018). Introspection of subjective feelings is sensitive and specific. *Journal of Experimental Psychology: Human Perception and Performance, 44*(2), 215.

Questienne, L., Van Opstal, F., van Dijck, J. P., & Gevers, W. (2018). Metacognition and cognitive control: behavioural adaptation requires conflict experience. *Quarterly Journal of experimental psychology, 71*(2), 411-423.

Rey-Mermet, A., & Gade, M. (2016). Contextual within-trial adaptation of cognitive control: Evidence from the combination of conflict tasks. *Journal of experimental psychology: human perception and performance, 42*(10), 1505.

Riesel, A., Klawohn, J., Kathmann, N., & Endrass, T. (2017). Conflict monitoring and adaptation as reflected by N2 amplitude in obsessive-compulsive disorder. *Psychological medicine, 47*(8), 1379.

Schiltenwolf, M., Kiesel, A., & Dignath, D. (2023). No temporal decay of cognitive control in the congruency sequence effect. *Journal of Experimental Psychology: Learning, Memory, and Cognition, 49*(8), 1247.

Schmidt, J. R., & De Houwer, J. (2011). Now you see it, now you don't: Controlling for contingencies and stimulus repetitions eliminates the Gratton effect. *Acta psychologica, 138*(1), 176-186.

Schmidt, J. R., & Weissman, D. H. (2014). Congruency sequence effects without feature integration or contingency learning confounds. *PLoS One, 9*(7), e102337.

Schmidt, J. R., & Weissman, D. H. (2015). Contingent attentional capture triggers the congruency sequence effect. *Acta Psychologica, 159*, 61-68.

Schroeder, P. A., Dignath, D., & Janczyk, M. (2018). Individual differences in uncertainty tolerance are not associated with cognitive control functions in the flanker task. *Experimental Psychology, 65*(4), 245.

Schuch, S., Philipp, A. M., Maulitz, L., & Koch, I. (2022). On the reliability of behavioral measures of cognitive control: retest reliability of task-inhibition effect, task-preparation effect, Stroop-like interference, and conflict adaptation effect. *Psychological research*, *86*(7), 2158-2184.

Shi, K., & Wang, L. (2022). The effect of irrelevant response dimension on stimulus response compatibility. *Acta Psychologica*, *223*, 103495.

Soutschek, A., & Schubert, T. (2013). Domain-specific control mechanisms for emotional and nonemotional conflict processing. *Cognition*, *126*(2), 234-245.

Spapé, M. M., & Hommel, B. (2014). Sequential modulations of the Simon effect depend on episodic retrieval. *Frontiers in Psychology*, *5*, 855.

Spapé, M. M., Band, G. P., & Hommel, B. (2011). Compatibility-sequence effects in the Simon task reflect episodic retrieval but not conflict adaptation: evidence from LRP and N2. *Biological Psychology, 88*(1), 116-123.

Spinelli, G., & Lupker, S. J. (2023). Robust evidence for proactive conflict adaptation in the proportion-congruent paradigm. *Journal of Experimental Psychology: Learning, Memory, and Cognition*, *49*(5), 675.

Steenbergen, H. V., Band, G. P., & Hommel, B. (2009). Reward counteracts conflict adaptation: Evidence for a role of affect in executive control. *Psychological Science*, *20*(12), 1473-1477.

Stürmer, B. (2011). Reward and punishment effects on error processing and conflict control. *Frontiers in psychology, 2*, 335.

Stürmer, B., Leuthold, H., Soetens, E., Schröter, H., & Sommer, W. (2002). Control over location-based response activation in the Simon task: behavioral and electrophysiological evidence. *Journal of Experimental Psychology: Human Perception and Performance, 28*(6), 1345.

Surrey, C., Kretschmer-Trendowicz, A., Altgassen, M., & Fischer, R. (2019). Contextual recruitment of cognitive control in preadolescent children and young adults. *Journal of experimental child psychology, 183*, 189-207.

Suzuki, K., & Shinoda, H. (2015). Transition from reactive control to proactive control across conflict adaptation: An sLORETA study. *Brain and cognition, 100*, 7-14.

Tang, D., Chen, X., Li, H., & Lei, Y. (2022). Distributional analyses reveal the individual differences in congruency sequence effect. *Plos one, 17*(8), e0272621.

Thibault, S., Hug, F., & Deschamps, T. (2019). Performance fatigability does not impact the inhibitory control. *Neuroscience research, 146*, 48-53.

Tomat, M., Wendt, M., & Jacobsen, T. (2023). Attentional adjustment in priming tasks: control strategies depend on context. *Cognitive Processing, 24*(1), 1-23.

Tomat, M., Wendt, M., Luna-Rodriguez, A., & Jacobsen, T. (2021). Adjustments of selective attention to response conflict–controlling for perceptual conflict, target-distractor identity, and congruency level sequence pertaining to the congruency sequence effect. *Attention, Perception, & Psychophysics, 83*(6), 2531-2550.

Torres-Quesada, M., Funes, M. J., & Lupiáñez, J. (2013). Dissociating proportion congruent and conflict adaptation effects in a Simon–Stroop procedure. *Acta psychologica, 142*(2), 203-210.

Ullsperger, M., Bylsma, L. M., & Botvinick, M. M. (2005). The conflict adaptation effect: It’s not just priming. *Cognitive, Affective, & Behavioral Neuroscience, 5*(4), 467-472.

van Bochove, M. E., Van der Haegen, L., Notebaert, W., & Verguts, T. (2013). Blinking predicts enhanced cognitive control. *Cognitive, Affective, & Behavioral Neuroscience, 13*(2), 346-354.

Van der Linden, L., Dricot, L., De Letter, M., Duyck, W., de Partz, M. P., Ivanoiu, A., & Szmalec, A. (2018). A case study about the interplay between language control and cognitive abilities in bilingual differential aphasia: Behavioral and brain correlates. *Journal of Neurolinguistics, 46*, 37-68.

van Driel, J., Swart, J. C., Egner, T., Ridderinkhof, K. R., & Cohen, M. X. (2015). (No) time for control: frontal theta dynamics reveal the cost of temporally guided conflict anticipation. *Cognitive, Affective, & Behavioral Neuroscience, 15*(4), 787-807.

Van Gaal, S., Lamme, V. A., & Ridderinkhof, K. R. (2010). Unconsciously triggered conflict adaptation. *PloS one, 5*(7), e11508.

van Steenbergen, H., Band, G. P., & Hommel, B. (2015). Does conflict help or hurt cognitive control? Initial evidence for an inverted U-shape relationship between perceived task difficulty and conflict adaptation. *Frontiers in Psychology, 6*, 974.

van Steenbergen, H., Weissman, D. H., Stein, D. J., Malcolm-Smith, S., & van Honk, J. (2017). More pain, more gain: Blocking the opioid system boosts adaptive cognitive control. *Psychoneuroendocrinology*, *80*, 99-103.

Verbruggen, F., Notebaert, W., Liefooghe, B., & Vandierendonck, A. (2006). Stimulus-and response-conflict-induced cognitive control in the flanker task. *Psychonomic Bulletin & Review, 13*(2), 328-333.

Wang, T., Chen, X., Pan, W., Xiao, Q., & Chen, A. (2021). The neural network underlying individual differences in conflict adaptation effect. *Biological Psychology, 164*, 108150.

Weissman, D. H. (2020). Interacting congruency effects in the hybrid Stroop–Simon task prevent conclusions regarding the domain specificity or generality of the congruency sequence effect. *Journal of Experimental Psychology: Learning, Memory, and Cognition, 46*(5), 945.

Weissman, D. H. (2019). Let your fingers do the walking: Finger force distinguishes competing accounts of the congruency sequence effect. *Psychonomic Bulletin & Review, 26*(5), 1619-1626.

Weissman, D. H., Colter, K. M., Grant, L. D., & Bissett, P. G. (2017). Identifying stimuli that cue multiple responses triggers the congruency sequence effect independent of response conflict. Journal of Experimental Psychology: *Human Perception and Performance, 43*(4), 677.

Weissman, D. H., Colter, K., Drake, B., & Morgan, C. (2015). The congruency sequence effect transfers across different response modes. *Acta psychologica, 161*, 86-94.

Weissman, D. H., Egner, T., Hawks, Z., & Link, J. (2015). The congruency sequence effect emerges when the distracter precedes the target. *Acta Psychologica, 156*, 8-21.

Weissman, D. H., Grant, L. D., & Jones, M. (2020). The congruency sequence effect in a modified prime-probe task indexes response-general control. *Journal of Experimental Psychology: Human Perception and Performance*, *46*(11), 1387.

Weissman, D. H., Hawks, Z. W., & Egner, T. (2016). Different levels of learning interact to shape the congruency sequence effect. *Journal of Experimental Psychology: Learning, Memory, and Cognition, 42*(4), 566.

Weissman, D. H., Jiang, J., & Egner, T. (2014). Determinants of congruency sequence effects without learning and memory confounds. *Journal of Experimental Psychology: Human Perception and Performance, 40*(5), 2022.

Weissman, D., & Carp, J. (2013). Congruency sequence effects are driven by previous-trial congruency, not previous-trial response conflict. *Frontiers in psychology, 4*, 587.

Weldon, R. B., Mushlin, H., Kim, B., & Sohn, M. H. (2013). The effect of working memory capacity on conflict monitoring. *Acta psychologica, 142*(1), 6-14.

Wendt, M., Kiesel, A., Geringswald, F., Purmann, S., & Fischer, R. (2014). Attentional adjustment to conflict strength. *Experimental Psychology, (61)*1.

Wendt, M., Kluwe, R. H., & Peters, A. (2006). Sequential modulations of interference evoked by processing task-irrelevant stimulus features. *Journal of Experimental Psychology: Human Perception and Performance, 32*(3), 644.

Wendt, M., Luna-Rodriguez, A., & Jacobsen, T. (2014). Sequential modulation of distractor-interference produced by semantic generalization of stimulus features. *Frontiers in psychology, 5*, 1271.

West, R., Bailey, K., Tiernan, B. N., Boonsuk, W., & Gilbert, S. (2012). The temporal dynamics of medial and lateral frontal neural activity related to proactive cognitive control. *Neuropsychologia, 50*(14), 3450-3460.

Whitehead, P. S., Brewer, G. A., & Blais, C. (2019). Are cognitive control processes reliable?. *Journal of experimental psychology: learning, memory, and cognition, 45*(5), 765.

Wühr, P. (2004). Sequential modulations of logical-recoding operations in the Simon task. *Experimental Psychology, 51*(2), 98.

Yang, G., Nan, W., Zheng, Y., Wu, H., Li, Q., & Liu, X. (2017). Distinct cognitive control mechanisms as revealed by modality-specific conflict adaptation effects. *Journal of experimental psychology: human perception and performance, 43*(4), 807.

Yang, G., Wang, K., Nan, W., Li, Q., Zheng, Y., Wu, H., & Liu, X. (2022). Distinct brain mechanisms for conflict adaptation within and across conflict types. *Journal of Cognitive Neuroscience, 34*(3), 445-460.

Zhang, M., Li, Q., Yin, S., & Chen, A. (2021). Changes in the level of conflict trigger conflict adaptation. *Acta Psychologica Sinica, 53*(2), 128.

Zhao, X., Jia, L., & Maes, J. H. (2018). Effect of achievement motivation on cognitive control adaptations. *Journal of Cognitive Psychology, 30*(4), 453-465.

Total number of papers included in the Meta-analysis: 146
